# Alkaline Extraction and Ethanol Precipitation of High-Molecular-Weight Xylan Compounds from *Eucalyptus* Residues

**DOI:** 10.3390/polym17121589

**Published:** 2025-06-06

**Authors:** María Noel Cabrera, Antonella Rossi, Juan Ignacio Guarino, Fernando Esteban Felissia, María Cristina Area

**Affiliations:** 1 Engineering School, Chemical Engineering Institute/Forest Processes Engineering Group, Universidad de la República, Montevideo 11300, Uruguay; 2 Institute of Materials of Misiones, Pulp and Paper Program (PROCYP), Universidad Nacional de Misiones, Posadas 3300, Misiones, Argentina; ffelissia@gmail.com

**Keywords:** eucalyptus wood, xylan, hemicelluloses extraction, antisolvent precipitation

## Abstract

Alkaline treatment is well suited for extracting high-molecular-weight hemicelluloses, specifically hardwoods xylans, which, due to their polymer structure and chemical characteristics, enable the production of films with desirable mechanical, barrier, and optical properties for packaging applications. Despite its relevance, the optimization of antisolvent addition has received little attention in the literature. This study explores the use of eucalyptus industrial residue as feedstock, utilizing a statistical design to determine the optimal extraction conditions for hemicelluloses while minimizing the lignin content in the recovered liquor. The process uses alkali loads that are compatible with those in conventional Kraft pulp mills. Optimal extraction conditions involve a temperature of 105 °C, 16.7% NaOH charge, and 45 min at maximum temperature. The resulting liquor was subjected to ethanol precipitation under varying pH conditions (initial pH, 9, 7, 5, and 2) and different ethanol-to-liquor ratios (1:1 to 4:1). The acidification was performed using hydrochloric, sulfuric, and acetic acids. Ethanol served as the main antisolvent, while isopropyl alcohol and dioxane were tested for comparison. Results show that 2.3 ± 0.2% of xylans (based on oven-dry biomass) could be extracted, minimizing lignin content in the liquor. This value corresponds to the extraction of 15.6% of the xylans present in the raw material. The highest xylan precipitation yield (78%) was obtained at pH 7, using hydrochloric acid for pH adjustment and an ethanol-to-liquor ratio of 1:1. These findings provide valuable insight into optimizing hemicellulose recovery through antisolvent precipitation, contributing to more efficient biomass valorization strategies within lignocellulosic biorefineries.

## 1. Introduction

The growing urgency of climate change has driven a global transition toward sustainable, resource-efficient economic models. Within this shift, the circular economy offers a compelling strategy by minimizing waste, encouraging material reuse, and closing production loops. A central element of this approach is the development of lignocellulosic biorefineries, which convert renewable biomass into a broad array of value-added products, such as biofuels, chemicals, and materials [1,2,3]. Lignocellulosic biomass, including agricultural residues, forest waste, and energy crops, is an abundant yet underutilized resource. Fully valorizing its components (cellulose, hemicelluloses, and lignin) is key to advancing industrial-scale bioprocessing. However, its complex and recalcitrant structure presents challenges for efficient conversion. Effective pretreatment is therefore essential to disrupt the lignocellulosic matrix, separate components, break hydrogen bonds, and increase fiber surface area for further processing [1,4].

Alkaline pretreatment is one of the most commonly used chemical methods for lignocellulosic biomass deconstruction. Besides their non-corrosive nature, they are usually recovered and reused, resulting in significant economic and environmental benefits compared with alternative chemical approaches [1,5,6]. During alkaline hydrolysis, fiber solvation induces swelling, and saponification of ester bonds disrupts polymer crosslinking, opening hemicellulose and lignin structures. Glycosidic bonds within hemicelluloses are cleaved, and acetyl and uronic groups are removed. Lignin depolymerization occurs via cleavage of α- and β-aryl ether linkages, further degrading its structure. Under harsher conditions, peeling reactions may occur in hemicelluloses and cellulose, decreasing the degree of polymerization [5,6,7,8].

Alkaline treatment is well suited for film applications, as it enables the extraction of relatively high-molecular-weight hemicelluloses fractions (over 10,000 Da). The film-forming ability of these polysaccharides depends on factors such as polymerization degree, structural heterogeneity, substituent type, and distribution, which influence the mechanical, barrier, and thermal properties of films. Xylans are the main polysaccharides in hardwood hemicelluloses. Xylan-based films offer low oxygen and aroma permeability and high light transmittance, making them promising for packaging applications [9,10].

Usually, pretreatment methods for deconstructing lignocellulosic biomass do not fully separate its components. Antisolvent precipitation is a widely applied technique to isolate and purify hemicelluloses, as hemicelluloses have much lower solubility in the antisolvent than in the aqueous phase [1,6,11]. Solvent precipitation involves adding a non-solvent (antisolvent) to a solution to gradually precipitate polysaccharides. Unlike the solvent that dissolves the target compound, the antisolvent induces separation by reducing solubility. This method uses commercially available solvents and cost-effective equipment, making it easily scalable and well-suited for polysaccharide fractionation.

Polysaccharides dissolve in aqueous solutions through hydrogen bonding with water molecules. Adding dehydrating agents like ethanol or isopropanol disrupts these bonds, causing precipitation. The solvent’s dielectric constant plays a key role. Lower values enhance intermolecular attractions, induce conformational changes, promote aggregation, and ultimately trigger polysaccharide precipitation [12].

Alcohols such as ethanol and isopropanol, with lower dielectric constants than water (80 F/m for water vs. 20–35 F/m for various alcohols at 25 °C), are widely used as antisolvents or precipitation agents. Polysaccharide precipitation is achieved by gradually adding a less polar solvent to a polar medium [12,13,14]. Ethanol is the most commonly used antisolvent for hemicelluloses, though methanol, isopropanol, acetone, and other water-miscible organic solvents are also employed. The polarity order (methanol > ethanol > isopropanol > acetone) generally correlates with precipitation efficiency: methanol < ethanol < isopropanol < acetone, though ethanol is preferred due to its safety, availability, and low cost [6,13,14,15].

Polysaccharides with different molecular weights and chemical structures exhibit varying solubilities. Beyond molecular weight, solubility differences arise from variations in sugar composition, side groups, linkage types, branching degree and pattern, and degree of substitution [11,12,14]. In the case of hemicelluloses, studies have shown that more-branched hemicelluloses require higher ethanol concentrations [13,16].

Working at low temperatures reduces solvent polarity and polysaccharide solubility, enhancing precipitation efficiency. As a result, it is common practice to incubate the system at 4 °C for 24 h or more after solvent addition to allow conformational changes and promote precipitation [1,11,15]. The pH of the aqueous solution also influences precipitation efficiency. At high pH, hydroxyl groups on the polysaccharide backbone may ionize, altering solubility. This effect is more pronounced in charged polysaccharides due to the ionization of carboxyl and amino groups [14,17]. Some studies have used pH values of 5.5 or 6 for non-solvent addition without a clear justification [11,13,16]. Additionally, the solubility of other solution components, such as lignin or phenolic compounds from lignocellulosic biomass, must be considered. Identifying the optimal pH is essential for efficiently precipitating polysaccharides and separating other constituents, as pH significantly affects lignin solubility more than hemicelluloses.

This study examines xylan extraction from industrial eucalyptus residues, optimizing key process variables (soda charge, temperature, and time) to maximize the recovery of high-molecular-weight xylan while minimizing lignin co-extraction. This work goes beyond traditional approaches by investigating the largely unexplored area of solvent precipitation parameters, mainly how pH and ethanol volume influence the process. It further investigates alternative acids (acetic and sulfuric) for pH adjustment and evaluates other solvents (dioxane, isopropanol) as substitutes for ethanol. The current literature does not justify selecting solvent addition parameters during hemicellulose precipitation. This study addresses that critical gap, providing novel insights that enhance the scientific understanding and practical application of hemicellulose recovery from lignocellulosic biomass.

## 2. Materials and Methods

### 2.1. Raw Material

*Eucalyptus* pinchips, a by-product from chip screening at a Kraft pulp mill (UPM, Fray Bentos, Uruguay), were used as raw material. Typically destined for biomass energy, the pin chips were received at ~45% moisture content and dried to 8% in a tray air-forced dryer at 40 °C. The particle size distribution was >3.36 mm (7.3%), 3.36–1.40 mm (36.6%), 1.40–1.19 mm (42.1%), 1.19–0.50 mm (10.3%), and <0.50 mm (3.6%). The fraction < 0.50 mm was excluded. Ethanol/water extractives were quantified following TAPPI T204 cm-07. Structural carbohydrates (glucan, xylan, arabinan), acid-soluble and insoluble lignin, acetyl groups, and ash were analyzed using modified NREL TP 510-42618 and NREL TP 510-42622 procedures. A Shimadzu Lab Solution HPLC (Kyoto, Japan) was used, equipped with refractive index detector (RID-10A) and UV–visible detectors, and an Aminex HPX-87H (BIORAD, Hercules, CA, USA) column at 35 °C, eluted with 5 mM H_2_SO_4_ at 0.6 mL/min. Soluble lignin was measured at 210 nm using a Shimadzu Mini 1240 UV–vis spectrophotometer (Kyoto, Japan) (ε = 110 L·g^−1^·cm^−1^). The composition is presented in Table 1.

### 2.2. Alkaline Extraction

A 3^3^ factorial experimental design was implemented, considering three key parameters: maximum extraction temperature (105, 130, and 155 °C), time at that temperature (45, 90, and 135 min), and alkali charge, expressed as the percentage of sodium hydroxide relative to the oven-dry wood mass (6.7, 11.7 and 16.7% NaOH/o.d. biomass). A liquid-to-solid ratio of 5 was maintained. All experiments were performed in duplicate. Parameter ranges were selected to align with a biorefinery framework, allowing subsequent valorization of cellulose and lignin. The alkali charges and treatment times employed in this study are of the same order of magnitude as those used in conventional pulp mills. Operating at lower temperatures with comparable alkali charges would insufficiently disrupt biomass structure, limiting hemicellulose extraction.

Data analysis was conducted using Statgraphics^®^ Centurion XVI (The Plains, VA, USA) including analysis of variance (ANOVA), response surface modeling, and optimization via the desirability function at a 95% confidence level. Coded values for the statistical analysis were obtained by normalizing individual response functions from −1 to +1 and subsequently integrated through a geometric mean to compute a composite desirability index, which is then maximized.

After each extraction, solids were separated by drainage and centrifugation (1000 rpm) and then washed with deionized water. Extraction solid yield was calculated as the percentage of dry mass loss relative to the initial weight. The solid fraction was reserved for potential glucan and lignin valorization, which was not covered in this study. Liquors were analyzed for pH, total solids (at 105 °C), and UV absorbance at 280 nm as an indicator of lignin and phenolic group content [18]. Total carbohydrates (quantified as monomeric sugars: xylose, glucose, and arabinose), organic acids (acetic, formic), and degradation products (furfural, hydroxymethylfurfural (HMF)) were quantified using a modified NREL TP-510-42623 method on an Aminex HPX-87H column (BIORAD, Hercules, CA, USA).

### 2.3. Antisolvents Precipitation

Various ethanol-based precipitation strategies were evaluated to optimize xylan recovery from the extraction liquor. Initially, the influence of the solution’s initial pH before ethanol addition was assessed. The pH was adjusted to different values—the initial pH of the liquor, 9, 7, 5, and 2—using hydrochloric acid. Following pH adjustment, samples were centrifuged at 3000× *g* for 20 min, and the resulting precipitate was collected. Precipitation yield was calculated relative to the total dissolved solids in the liquor.

**Figure 1:**
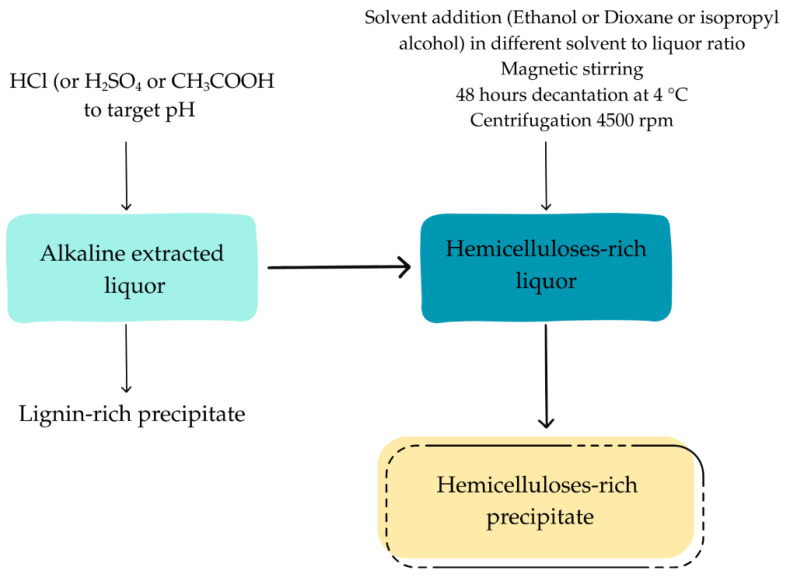
Tests for the precipitation of hemicelluloses extracted with sodium hydroxide (alkaline extracted liquor).

Ethanol was subsequently added to the supernatant at a ratio of two volumes of ethanol per volume of liquor (2:1 (*v*/*v*)) with constant magnetic stirring, followed by an additional 30 min stirring period. The mixture was allowed to decant at 4 °C for 48 h. The precipitate was washed with ethanol and dried at 37 °C to a constant weight. This protocol was applied across all tested conditions, each performed in duplicate (see Figure 1).

Different ratios, ranging from 1:1 to 4:1, were tested to investigate the effect of the ethanol-to-liquor volume ratio on hemicellulose precipitation. For this assay, the liquor pH was initially adjusted to 7 before ethanol addition. In subsequent tests, sulfuric and acetic acids—both industrially relevant—were employed to evaluate the effect of acid type on precipitation efficiency. Finally, to assess the behavior of xylosaccharides with alternative antisolvents, dioxane and isopropanol were used instead of ethanol. Each was added at a 2:1 solvent-to-liquor volume ratio, either at the liquor’s original pH or adjusted to pH 7 using hydrochloric acid.

The precipitation yields were evaluated by calculating the ratio between the dry mass of the precipitate and the initial dissolved solids content of the extracted liquor. In the liquid fraction (after pH adjustment), carbohydrates and organic acids (acetic and formic), degradation products (HMF and furfural), and the absorbance at 280 nm were analyzed. To determine carbohydrates, organic acids, and the absorbance at 280 nm in the precipitates, samples were dissolved in 0.1 M NaOH before measurement using the previously described protocols. Molecular weight distribution and ash content were determined for selected precipitates after dissolution in 0.1 M NaOH. Molecular weight distribution was analyzed by high-performance size-exclusion chromatography (HPLC-SEC) with refractive index detection (RID) (Shimadzu Co., Kyoto, Japan). Samples were filtered through 0.45 and 0.22 µm filters and injected into an HPLC system equipped with three MCX 1000 Å and two MCX 100 Å PSS^®^ columns (Mainz, Germany). The mobile phase consisted of a phosphate buffer (pH 12; 6.9 g NaH₂PO₄·H₂O and 3.2 g NaOH per liter, pH adjusted with NaOH), operated at 35 °C and 0.6 mL/min. Calibration was performed using PSS^®^ sodium polystyrene sulfonate standards (Mainz, Germany) with molecular weights of 4230, 7930, 10,600, 14,900, 20,700, 29,100, and 64,100 Da.

The precipitates were analyzed using FTIR-ATR spectroscopy using a Shimadzu IR Affinity 1-S FTIR (Kyoto, Japan) spectrometer equipped with a Pike MIRacle ATR accessory (1.8 mm diamond/ZnSe crystal). Spectra were collected in the 4000–600 cm^−1^ range at 4 cm^−1^ resolution, averaging 32 scans per sample.

## 3. Results and Discussion

### 3.1. Alkaline Extraction

Alkaline treatment enabled the selective extraction of xylosaccharides fractions from eucalyptus wood residues, accompanied by partial lignin removal. Table 2 summarizes the extraction yield, xylosaccharides content, and absorbance at 280 nm—used as an indirect indicator of lignin-derived compounds—under various experimental conditions. All liquors exhibited final pH values above neutrality. Notably, those obtained at higher temperatures (155 °C) and lower NaOH charge (6.7%) showed pH values closest to 7, irrespective of extraction time. The observed pH decrease during extraction is attributed to the release of acetyl and uronic substituents from hemicellulose, the formation of hydroxy acids via alkaline peeling reactions, and the neutralization of fatty acids from extractives [7,19].

At a given NaOH charge, extraction yield increased with temperature. Similarly, increasing the alkali charge at constant temperature enhanced the yield. A comparable, though less pronounced, trend was observed with extraction time. As established, alkaline treatment induces polysaccharide degradation via several mechanisms, including “peeling” reactions—where terminal monosaccharides are cleaved from polysaccharide chains, forming hydroxy acids—and alkaline hydrolysis of glycosidic bonds, which generates alkali-labile reducing ends susceptible to secondary peeling. Concurrently, hemicellulose deacetylation, lignin solubilization and degradation, and intensive removal of extractives occur, along with ester saponification [1,7]. These reactions are less prominent at lower temperatures but become increasingly significant at elevated temperatures, substantially altering the composition of the extracted material [19].

The highest xylosaccharide concentration was obtained under the most severe conditions, reaching 2.5% (based on oven-dry biomass) in the liquor, corresponding to 17% of the initial xylan content.

Absorbance at 280 nm reached its highest values in liquors produced under high alkali charge and elevated temperature. In contrast, despite a high alkali charge, absorbance dropped considerably at lower temperatures, consistent with the limited extent of delignification below 130 °C [19,20]. Notably, absorbance values under high temperature and low alkali charge were comparable to those obtained at low temperature and high alkali charge.

#### Statistical Treatment

The subsequent models describe the response variables associated with the alkaline extraction treatment (Equations (1)–(3)). Only those factors demonstrating statistical significance (*p* < 0.05) have been retained in the equations. The coefficients are expressed using real variables, such that their magnitudes denote the relative contribution of each factor to the model.

The factors significantly affecting the extraction yield are A: NaOH charge (NaOH); B: temperature (T); and C: time (t) and the AA (quadratic effect of NaOH charge) and AB (interaction between NaOH charge and temperature) interactions. The ANOVA for the xylosaccharides content in the liquor showed that three effects (A: NaOH charge; B: temperature; and AB interaction) were significantly different, with a confidence level of 95.0%. According to the interaction of the variables, the soda charge is a very important factor at low temperatures but loses significance as the temperature increases. For the absorbance of the extraction liquor at 280 nm (diluted 1/1500), the ANOVA results indicate that three effects were statistically significant: A (NaOH charge), B (temperature), and their interaction (AB). It is noteworthy that these same factors also exert a significant influence on the concentration of xylosaccharides in the liquor.
Yield (%) = 22.7 + 5.9 × NaOH + 4.8. × T + 1.8 × t − 1.9 × NaOH^2^ + 2.4 × NaOH × TR^2^ = 93.7%(1)Xylosaccharides (%) = 1.8 + 0.4 × NaOH + 0.4 × T − 0.2 × NaOH × TR^2^ = 88.9%(2)Absorbance 280 nm = 0.63 + 0.16 × T + 0.18 × NaOH + 0.11 × T × NaOHR^2^ = 92.1%(3)

**Figure 2:**
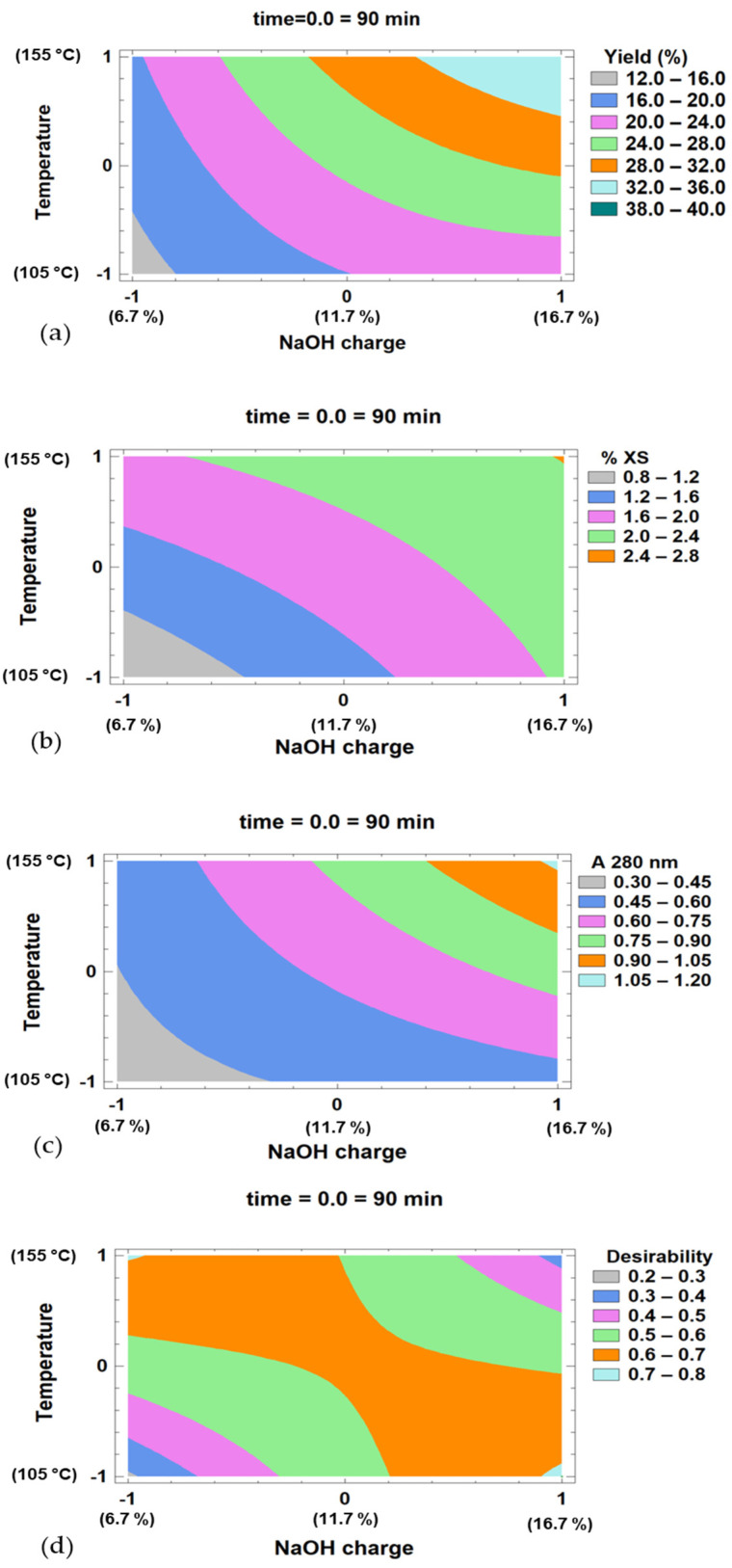
(**a**–**c**). Estimated surface response contour for the different responses’ models, illustrating the relationship between temperature, NaOH charge, and the response variable studies, for a constant time (90 min): (**a**) Extraction yield (%), (**b**): xylosaccharides content in the extracted liquor (% XS/o.d. biomass), (**c**) absorbance at 280 nm of the extracted liquor. (**d**) Contour curves of the response surface obtained by evaluating multiple responses: maximization of xylosaccharides present in the liquor and minimization of absorbance at 280 nm.

The estimated surface response contour plot for the extraction yield, xylosaccharides content in the liquor, and absorbance at 280 nm responses model are presented in Figure 2a–c. In addition, for the optimization of multiple responses of the experimental design, the desirability function was defined to maximize the xylosaccharides concentration in the liquor and minimize the absorbance at 280 nm. Figure 2d shows the obtained response.

The highest predicted desirability (0.711) was achieved under conditions of maximum NaOH charge (16.7%) and minimum temperature (105 °C), corresponding to coded variables (1, −1), with no significant effect of extraction time. Under these conditions, a predicted desirability of 0.711 is obtained for three times tested. In the extraction performed at 105 °C, 16.7% NaOH and 45 min, 2.3 ± 0.2% of xylosaccharides (o.d.w.) were extracted, representing 15.6 ± 0.3% of the xylosaccharides present in the initial wood.

A similarly high desirability (0.706) was observed at low NaOH charge (6.7%) and high temperature (155 °C), corresponding to coded variables (−1, 1), for the three times tested.

This analysis focuses on results obtained from hardwoods, primarily short-fiber species, to ensure meaningful comparisons. Several authors have employed alkali charges similar to those used in the pulp and paper industry, as in the present study, yielding comparable hemicellulose extraction outcomes. Van Heiningen et al. [21] processed a mixture of short-fiber wood chips (red oak, red maple, poplar, and southern magnolia) at 120–165 °C with NaOH charges ranging from 3% to 20% (as Na₂O) for 45–90 min. Reported extraction yields ranged from 8% to 40%, comparable to or slightly lower than those in this work. However, their xylosaccharide extraction was notably higher, with up to 54% xylan removal under conditions of 150 °C, 90 min, and 20% Na₂O (equivalent to 12.9% NaOH). This may be attributed to the higher hemicellulose content in cold-climate wood species compared with those from milder regions [7,21].

Lehto and Alén [7], working with birch chips at 130–150 °C and 1–8% NaOH (based on dry wood) for 30–90 min, achieved extraction yields ranging from 2% under mild to 16.5% under severe conditions (150 °C, 120 min, 8% NaOH). These results are comparable to the 19.4% yield observed in the present study at 155 °C, 135 min, and 6.7% NaOH. Under their most severe conditions, a 4% carbohydrate extraction was reported, though the xylosaccharide fraction was not specified. Given the lower xylan content in *Eucalyptus* spp. compared with birch (21.1% as xylose, dry basis), the extent of xylosaccharide recovery is considered comparable.

Morais de Carvalho et al. [22] conducted alkaline extraction on *Eucalyptus urophylla* and *Eucalyptus grandis* chips at 175 °C for 15 min (liquor-to-wood ratio 2:1) using NaOH charges of 5%, 10%, and 15%. They reported overall extraction yields of 5%, 12%, and 23%, respectively, which are lower than those achieved in this study. The higher yields here may be partly due to the use of pinchips—smaller than industrial chips—allowing enhanced alkali penetration and reactivity [2]. In addition, differences in whether wash waters were included in mass balance calculations may explain discrepancies, as yield is gravimetrically determined based on dry mass loss. Reported hemicellulose extraction ranged from 9.6% to 12.4% of non-glucose sugars (dry basis), corresponding to 47–60% removal of initial non-glucose carbohydrates.

Other studies [23,24,25,26] have utilized significantly higher alkali concentrations (40–300%, g alkali/g dry wood) at lower temperatures (40–125 °C), achieving xylan removal efficiencies of 40–90%. However, such conditions are impractical for industrial applications due to the need for efficient alkali recovery systems. Moreover, separating high concentrations of alkali and acid during neutralization complicates the recovery of hemicellulose precipitates after antisolvent addition [26].

Based on these comparisons, the selected conditions for further work were 105 °C, 45 min, and a 16.7% NaOH charge.

### 3.2. Ethanol Precipitation

#### 3.2.1. Ethanol Precipitation at Different Liquor pH Levels

**Figure 3:**
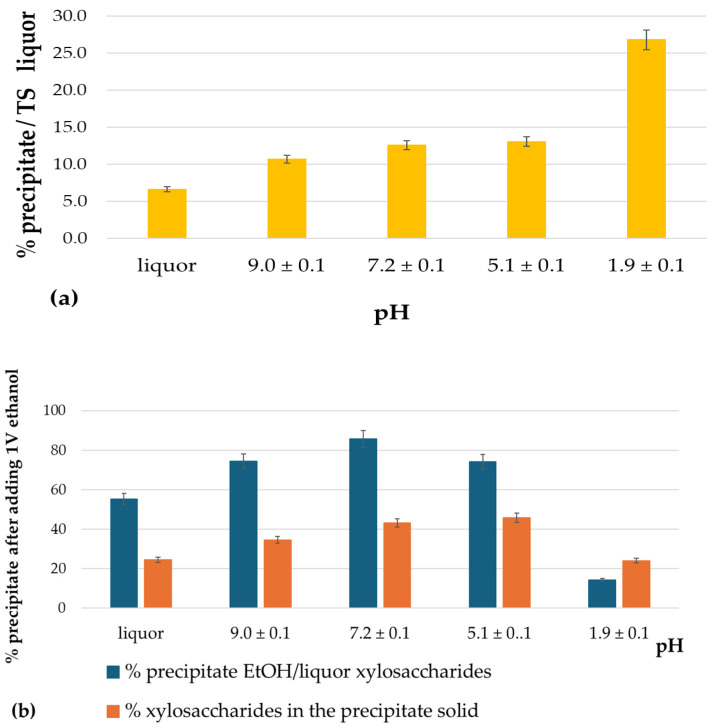
(**a**) Solids precipitated after adjusting the pH of the extracted liquor. (**b**) Precipitates after adding two volumes of ethanol per volume of liquor at different pHs. The orange bars show the percentage of xylosaccharides in each precipitate.

The pH of the alkaline-extracted liquor (105 °C, 45 min, with a 16.7% alkali charge) reached 12.4 ± 0.1. As previously mentioned, to obtain xylans with lower levels of contamination, the first step before the addition of the antisolvent is to reduce the pH in order to precipitate lignin-derived compounds. In this study, five pH conditions were evaluated: the original liquor pH and adjusted values of 9, 7, 5, and 2. Figure 3a presents the percentage of total precipitated solids obtained following pH adjustment at each level.

The percentage of precipitated material increased as the pH decreased, reaching a plateau at pH 7. The proportions of material precipitated at pH 7 and pH 5 were statistically equivalent at α = 0.05. However, a significant increase was observed when the pH was further reduced to 2, with the percentage of precipitated solids reaching 27%. The absorbance at 280 nm of the remaining liquor (after pH adjustment, precipitate separation, and considering a 1:500 dilution) decreased by 15% at pH 9 and 7, by 20% at pH 5, and by 60% at pH 2.

Regarding the xylosaccharides content in the liquor after pH adjustment, approximately 35% of the xylosaccharides precipitated at pH 9 and 7. This value increased to ~46% at pH 5 and 2.

Subsequently, ethanol was added to the pH-adjusted liquors, and the solutions were allowed to stand at 4 °C for two days. The resulting precipitates were recovered by centrifugation, washed with ethanol, and oven-dried at 37 °C for further analysis. The precipitates obtained at the four highest pH values exhibited similar visual characteristics, black with no discernible differences. In contrast, the precipitate formed at pH 2 had a slightly lighter brown hue, more noticeable in the wet state.

Figure 3b illustrates the percentage of xylosaccharides precipitated upon ethanol addition (relative to their content in the liquor before pH adjustment) and the xylosaccharides content in the resulting precipitates. Lowering the pH to 7.2 enhanced ethanol-induced precipitation, yielding up to 86 ± 4% of the xylosaccharides from the pH-adjusted liquor. No further improvement was observed at lower pH values. At pH 1.9, only ~15% of xylosaccharides were recovered by ethanol precipitation. On the other hand, when ethanol was added to the extract at the original pH, 24.6 ± 1.2% of the obtained precipitate was composed of xylosaccharides. This proportion increased to 43.1 ± 2.2% and 45.9 ± 2.3% at pH 7.2 and 5.1, respectively. At pH 1.9, the xylosaccharides content in the precipitate dropped to ~25%, with ash becoming the dominant component, even after washing the solids with ethanol before chemical analysis.

As noted in the introduction, several studies have reported successful xylan precipitation following pH adjustment to values between 5.5 and 6.5, using acetic or hydrochloric acid [11,27,28,29,30]. However, these works often lack a clear rationale for selecting this specific pH range. Even one of the earliest reports—O’Dwyer (1926)—described the precipitation of hemicelluloses from beech wood alkaline extracts using a “substantial amount of hydrochloric acid” followed by the addition of two volumes of ethanol [31]. According to the study, a significant portion of high-molecular-weight hemicelluloses (“Hemicelluloses A”) precipitated upon acidification, while the addition of ethanol resulted in a smaller, distinct precipitate. Despite this, the literature offers limited justification for selecting the 5.5–6.5 pH range.

**Figure 4:**
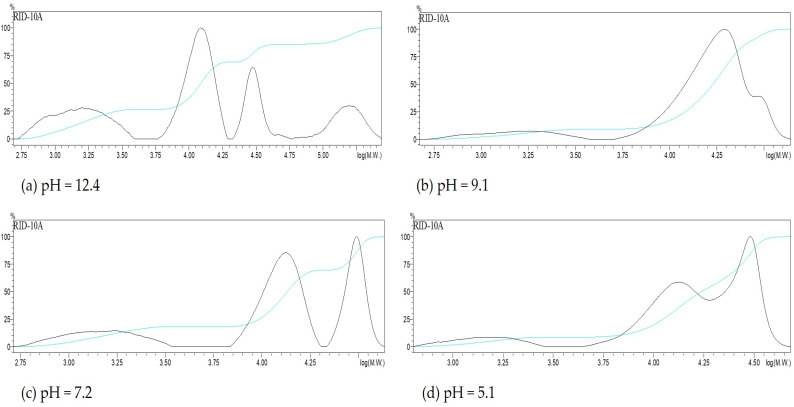
Molecular weight distribution of the precipitates obtained after adding ethanol at different pH. The light blue line shows the accumulative molecular weight. (**a**) precipitated obtained after adding ethanol at pH = 12.4, (**b**) precipitated obtained after adding ethanol at pH = 9.1, (**c**) precipitated obtained after adding ethanol at pH = 7.2 and (**d**) precipitated obtained after adding ethanol at pH = 5.1.

The molecular weight distributions of the obtained precipitates are shown in Figure 4. Table 3 summarizes the number-average (Mn) and weight-average (Mw) molecular weights, along with the relative area percentages of each distribution peak.

According to Table 3 and Figure 4, the Mw of the precipitate obtained by ethanol precipitation at the initial extract pH (12.4) was 33,243 ± 112 Da. For the other pH levels tested, Mw ranged between 16,800 and 26,600 Da. A peak at 154,000 Da was observed at pH 12.4, absent in the other cases, and likely corresponds to large lignin–carbohydrate complex (LCC) fragments that are removed by pH reduction. Similar high-molecular-weight fractions in xylan precipitates have been previously reported [32,33]. As pH decreases, the dominant peak shifts to the 18,000–28,000 Da region, while the low-molecular-weight peak (1300–1420 Da) diminishes or even disappears at pH 1.9.

In comparison, Testova et al. [23] reported an Mw of 19,800 Da for liquor extracted from birch chips at 95 °C with 2.5 M NaOH for 60 min. Vena et al. [24], using *Eucalyptus grandis* chips treated at 40 °C for 240 min with 2 M NaOH (liquor-to-wood ratio 4:1), obtained an Mw of 53,400 Da. The latter study used dialysis for precipitation and noted that lignin fragments may affect molecular weight estimates. Besides molecular weight, parameters such as linkage type, degree of polymerization, and substitution pattern significantly affect solubility and precipitation behavior [11,14].

**Figure 5:**
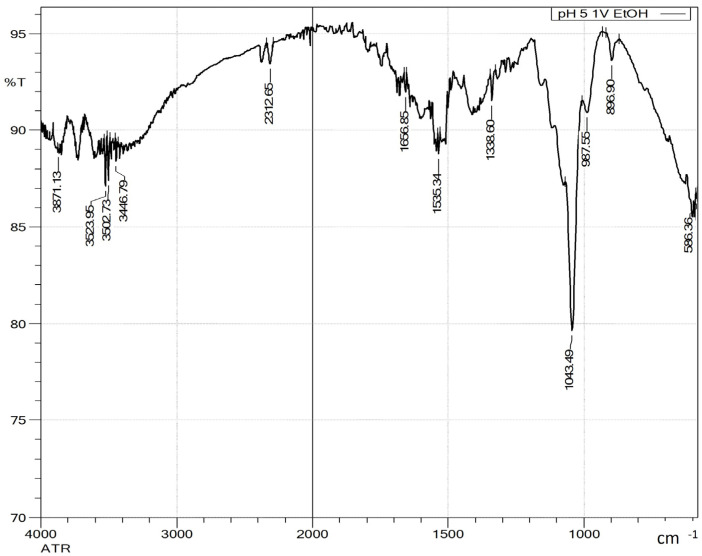
FTIR-ATR spectra of the precipitate obtained by adding one volume of ethanol at pH 5, indicating the wavenumbers of the characteristic peaks.

Figure 5 shows the FTIR-ATR spectra of the precipitates obtained after ethanol addition at pH 5. The spectra for other pH levels were qualitatively similar.

All precipitates exhibited absorption bands near 1040 cm^−1^, attributed to C–C and C–O stretching and C–OH bending vibrations in (1→4)-β-xylans. Bands between 1130–1160 cm^−1^, corresponding to C–O–C stretching (glycosidic linkages), and a characteristic peak at 895 cm^−1^, associated with C–H bending in the furan ring of β-glycosidic bonds, confirmed the presence of xylosaccharides [30,34,35,36].

A broad absorption in the 3300–3500 cm^−1^ range, corresponding to O–H stretching, was also observed [35,36]. Bands between 1465 and 1242 cm^−1^, assigned to C=O and C–O–R stretching, were associated with methylglucuronic acid and acetyl groups in the precipitates [30]. This band was more intense at pH 12.4 and diminished at lower pH values. C–H stretching vibrations (CH₃, CH₂, CH) in the 2988–2862 cm^−1^ region were only visible at pH 5 [35].

Aromatic ring vibrations at ~1512 cm^−1^, characteristic of phenolic compounds, were detected at pH 5 and 7 but not at pH 12.4, likely masked by the strong signal from methylglucuronic and acetyl groups. Conversely, band intensities in the 1600–1730 cm^−1^ region, related to C=C and C–H vibrations of lignin fragments, were markedly higher at pH 12.4. These findings suggest that the extracted polysaccharides were linked to LCCs, which are known to contain stable covalent bonds that are difficult to cleave [37,38].

The spectra at pH 9, 7, and 5 were very similar, with pH 7 yielding the highest xylosaccharide content in the precipitate. Based on these results, pH 7 appears optimal for lignin removal and xylosaccharide precipitation. Further lowering the pH to 5 showed no improvement, while reducing it to pH 2 significantly increased ash content and reduced the xylosaccharide fraction in the precipitate.

#### 3.2.2. Ethanol-to-Liquor Volume Ratio

**Figure 6:**
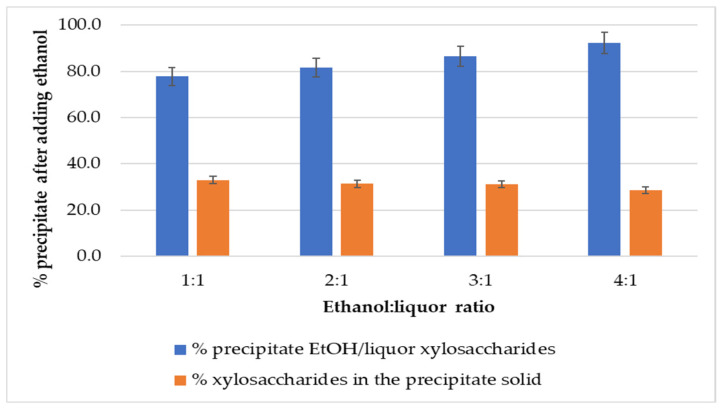
Obtained precipitates by adding different volumes of ethanol at pH 7 of the liquor.

Figure 6 presents the results obtained by adding ethanol at different volume ratios.

All of the precipitates obtained were black, like those obtained in the previous section. One volume of ethanol addition produced the precipitation of 78% of the xylosaccharides initially present in the liquor. As the ethanol volume increased, the precipitation percentage also increased, reaching 92% of xylosaccharides with four volumes of ethanol; these results are consistent with those reported in the literature [1,11,13,39].

Regarding the composition of the precipitate, approximately 30% consisted of xylosaccharides, with this value slightly decreasing as the ethanol-to-liquor ratio increased (from 33% to 28%). The literature revealed the use of various ethanol-to-liquor ratios for hemicellulose precipitation [1], ranging from ratios below 1 volume of ethanol [13,30], one volume [40], two volumes [31,38], three volumes [39,41], four volumes [16], and so on. There is a consensus that the hydrogen bonds maintaining polysaccharides soluble in aqueous media are rearranged upon the ethanol addition to the solution, leading to their precipitation [1,16,42]. However, none of the above studies justified the selection of the precipitation conditions.

**Figure 7:**
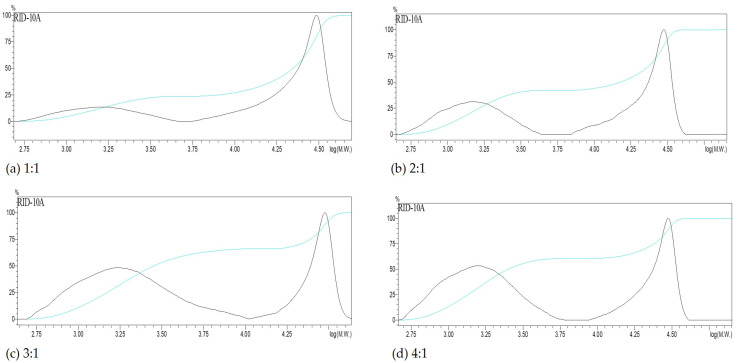
Molecular weight distribution of the precipitates obtained using different ethanol-to-liquor volume ratios at pH 7. The light blue line shows the accumulative molecular weight. (**a**) precipitated obtained at 1:1 ethanol-to-liquor volume ratio, (**b**) precipitated obtained at 2:1 ethanol-to-liquor volume ratio, (**c**) precipitated obtained at 3:1 ethanol-to-liquor volume ratio, and (**d**) precipitated obtained at 4:1 ethanol-to-liquor volume ratio.

The molecular weight distribution analysis (Figure 7 and Table 4) shows that the 1:1 ethanol-to-liquor volume ratio produced the highest weight-average molecular weight (Mw).

The decreasing xylosaccharides content in the precipitate with increasing ethanol volumes suggests a molecular weight-dependent precipitation mechanism. High-molecular-weight xylans tend to precipitate at lower ethanol concentrations, while lower-molecular-weight fractions remain in solution until higher ethanol concentrations are used. Consistently, molecular weight analysis of the precipitates revealed two distinct peaks: one in the 21,000–26,000 Da range and another around 1300–1400 Da. As the ethanol-to-liquor ratio increased, the relative area of the lower molecular weight peak became more prominent.

These results align with observations by Xu et al. [43], who worked with dextran and pullulan standards, and with findings by Bian [44], who studied stepwise ethanol precipitation using concentrations ranging from 10% to 90%. Both studies concluded that less-branched, high-molecular-weight hemicelluloses precipitate at lower ethanol concentrations, whereas more highly branched, lower-molecular-weight fractions require higher ethanol levels for precipitation.

**Figure 8:**
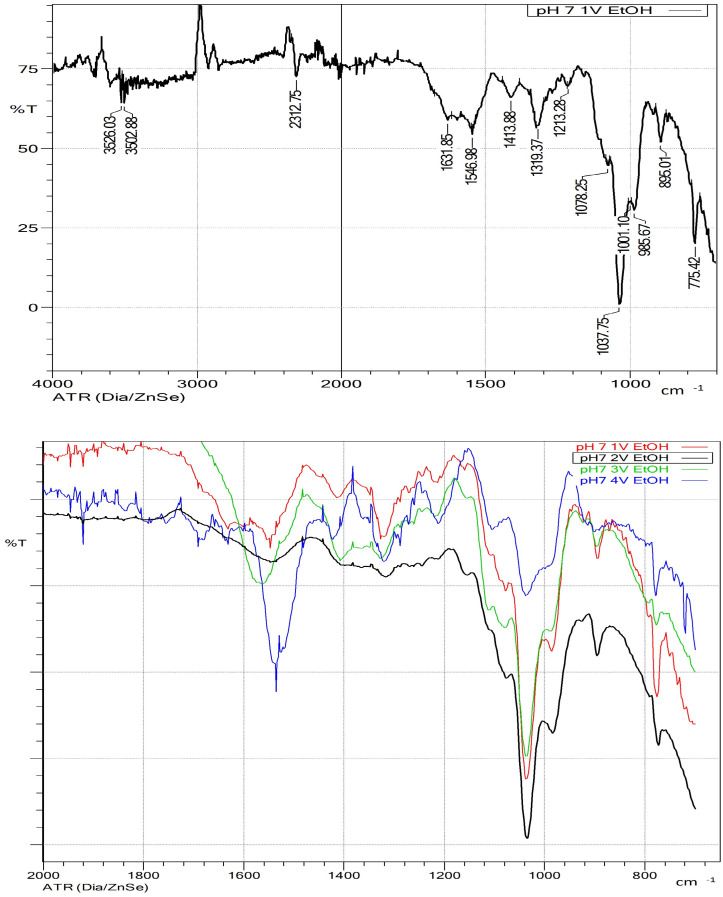
FTIR-ATR spectra of the obtained precipitates using different ethanol-to-liquor volume ratios at pH 7. At the top, the spectrum of the precipitate obtained by adding one volume of ethanol indicates the wavenumbers of the characteristic peaks. At the bottom, a comparative image of the spectra of the precipitates obtained with the different volumes used is presented (in this case, only the region between 2000–600 cm^−1^). Ethanol: liquor volume ratio: Red: 1:1, black: 2:1, green: 3:1, blue: 4:1.

Analyzing the FTIR-ATR spectrum of the precipitate obtained by adding ethanol in a 1:1 ratio (Figure 8), the characteristic bands corresponding to xylosaccharides can be observed (a band at approximately 1040 cm^−1^ assigned to (1–4)-β-xylans, and a band at 895 cm^−1^ corresponding to the β-glycosidic linkage). However, no bands are observed in the 3300–3500 cm^−1^ region, possibly corresponding to OH groups. Bands corresponding to methylglucuronic acids and acetyl groups are also present at 1212 cm^−1^ and 1413 cm^−1^, respectively.

The precipitates obtained after the addition of two and three volumes of ethanol exhibited FTIR-ATR spectra similar to those obtained with a 1:1 ethanol-to-liquor ratio. However, the intensity of the aromatic ring vibrations in the 1500–1560 cm^−1^ region—attributed to phenolic compounds—increased with ethanol concentration. This band became particularly prominent in the spectrum obtained following the addition of four volumes of ethanol, where it overshadowed the characteristic xylosaccharides bands. This behavior suggests that increasing ethanol concentration promotes the co-precipitation of lignin-derived fragments.

These findings indicate that a 1:1 ethanol-to-liquor volume ratio provides the most favorable condition for selective xylosaccharides precipitation. Although this ratio yields a slightly lower overall xylosaccharide recovery (78%) compared with the 4:1 ratio (92%), the resulting precipitate contains a higher proportion of xylosaccharides and exhibits greater molecular weight. This characteristic is particularly advantageous for applications such as biofilm formation [9]. Furthermore, reduced ethanol consumption under these conditions enhances process efficiency and supports a more sustainable process strategy.

#### 3.2.3. pH Adjustment Using Sulfuric and Acetic Acids Followed by Ethanol-Induced Precipitation

**Figure 9:**
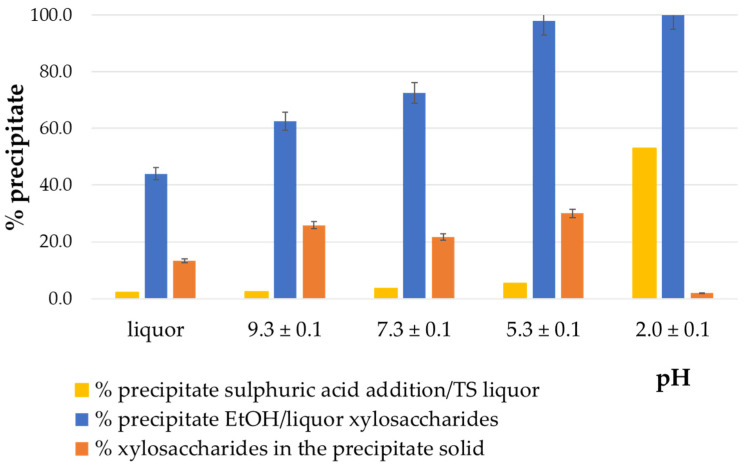
Yellow bars: percentage of the precipitated solids obtained by adjusting the pH of the extracted liquor with sulfuric acid. Blue bars: % percentage precipitated after adding 1 volume of ethanol to the liquor at different pH. Orange bars: percentage of xylosaccharides in the precipitated solids after adding 1 volume of ethanol.

Due to the corrosive nature of hydrochloric acid, its industrial use poses operational and material challenges. As a result, alternative acids commonly employed in industrial applications and cited in literature were evaluated for their suitability in pH adjustment before ethanol precipitation. Sulfuric acid, widely used for lignin precipitation from black liquor in the pulp and paper industry, was one of the alternatives considered [45,46]. Additionally, acetic acid was assessed as a more environmentally friendly alternative [47]. Figure 9 presents the yield and characteristics of the precipitated solids obtained after pH adjustment using sulfuric or acetic acid, followed by the addition of one volume of ethanol at different pH levels.

As with hydrochloric acid, the percentage of precipitated solids using sulfuric acid increased as the pH decreased. However, at pH levels above 5, hydrochloric acid induced a greater degree of precipitation. When the pH was lowered to approximately 2 using sulfuric acid, more than 50% of the initially dissolved solids precipitated, and the absorbance at 280 nm decreased by 57%, indicating substantial lignin removal. At this pH, 68% of the xylosaccharides originally present in the liquor were also removed. The precipitates obtained at pH 9, 7, and 5 exhibited similar characteristics to those obtained when using hydrochloric acid.

After ethanol addition, at the liquor’s initial pH, 44% of the xylosaccharides precipitated, and this value increased as the pH decreased. At pH 5.3, 84% of the initial xylosaccharides were recovered in the precipitate. Complete xylosaccharide removal was achieved at pH 2.0 when considering the combined precipitates obtained after pH adjustment and subsequent ethanol addition. These recovery values are higher than those obtained using hydrochloric acid. Furthermore, the precipitate formed after ethanol addition at pH 2 displayed distinct characteristics, notably a yellowish coloration.

In terms of composition, 30.4 ± 1.2% of the precipitate obtained at pH 5 (after ethanol addition) consisted of xylosaccharides, compared with 26.3 ± 1.0% at pH 9. However, only 2% of the precipitate obtained at pH 2 was composed of xylosaccharides, with ash content reaching 52%, indicating significant inorganic salt precipitation. The xylosaccharide contents in these precipitates were slightly lower than those obtained after pH adjustment with hydrochloric acid. In particular, the very low xylosaccharide percentage in the pH 2 precipitate can be attributed to the high proportion of inorganic matter, primarily sodium sulfate.

**Figure 10:**
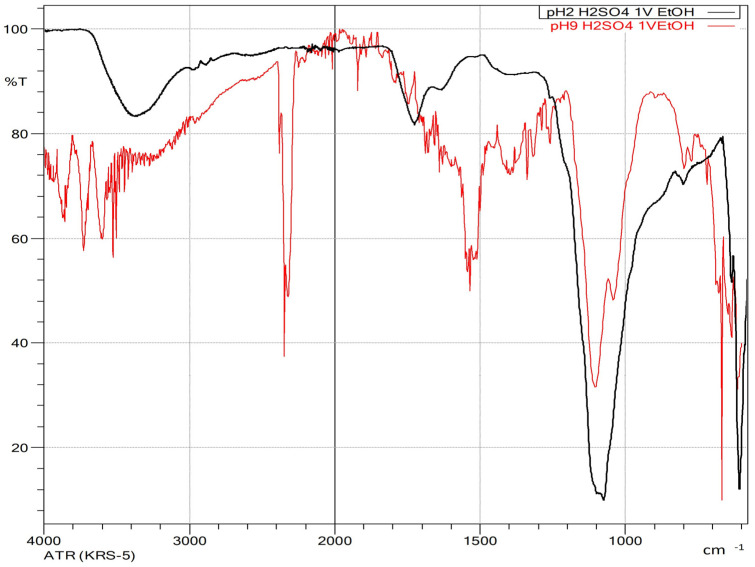
FTIR-ATR spectra of the precipitates by adding one volume of ethanol at different pHs—9 (red) and pH = 2 (black)—with the pH adjusted with sulfuric acid.

FTIR-ATR analysis (Figure 10) showed that the precipitate obtained at the liquor’s initial pH had higher lignin content than those obtained at other pH values, as evidenced by the increased intensity of peaks near 1510 cm^−1^ and in the 1600–1730 cm^−1^ region. The characteristic band associated with the stretching and bending vibrations of (1→4)-β-xylans appeared around 1040 cm^−1^. As the pH decreased, the intensity of lignin-associated peaks diminished markedly. However, the xylan band became increasingly obscured by the emergence of a new band near 1100 cm^−1^, attributed to sodium sulfate. At pH 5, this band intensified significantly, masking the xylan signal. This effect is consistent with the low solubility of sodium sulfate in ethanol (0.44 g/100 g solvent [48].

When acetic acid was used as the acidifying agent, 45% of the dissolved solids precipitated at pH 9 and 58% at pH 5. After drying, the resulting solids formed a black, gel-like film, potentially due to lignin acetylation. Xylosaccharide content in the supernatant could not be determined due to chromatographic interference from the acetic acid peak, which obscured signals in the relevant region. Peng et al. [13] adjusted the pH to 5.5 using acetic acid, which resulted in a “Hemicelluloses A” precipitate. However, their sample had undergone prior delignification with sodium chlorite, making direct comparisons unfeasible. FTIR-ATR spectra of solids precipitated with ethanol at pH 9 and 5 after acetic acid adjustment were very similar, but characteristic xylosaccharides peaks were not identifiable.

In summary, among the acids tested, hydrochloric acid yielded the most favorable results for adjusting the pH of the extracted liquor, particularly when considering the recovery rates and purity of the xylosaccharide fractions precipitated after ethanol addition.

### 3.3. Precipitation at Different pH Using Dioxane or Isopropanol as Antisolvent

Using dioxane as an antisolvent resulted in the precipitation of 30% of the xylosaccharides at the liquor’s initial pH and 21% at pH 7. In both cases, the xylosaccharide content in the precipitates was approximately 49 ± 1%. These values are comparable to those obtained with ethanol under similar conditions. However, considering dioxane’s higher cost and lower environmental compatibility, its use is not advantageous.

Santiago and Pascoal Neto [49], using black liquor from Kraft pulping of *Eucalyptus globulus*, precipitated hemicelluloses with two volumes of dioxane, followed by pH adjustment to 5 with acetic acid. They report that 78–84% of the polysaccharides in the precipitate were xylosaccharides, though they do not provide a full compositional analysis or describe the physical appearance of the precipitate. Similarly, Costabel [50], working with birch liquor extracted at 95 °C for 60 min in 2.5 M NaOH and precipitating with two volumes of dioxane followed by acetic acid adjustment to pH 5, reported that 63% of the resulting precipitate was xylosaccharides and 23% lignin, but again, no physical description was given.

Black stickies were formed at both pH levels when using isopropanol as an antisolvent, making it impossible to quantify the precipitation yields and the precipitate characteristics due to the sticky behavior of the solid.

## 4. Conclusions

Alkaline treatment enabled the removal of a fraction of xylosaccharides from eucalyptus wood residues. By optimizing the experimental conditions, considering both maximum xylosaccharide extraction and minimal lignin content in the liquor, the optimal parameters were found to be a temperature of 105 °C and a soda charge of 16.7% (o.d.w.). Extraction time, ranging from 45 to 135 min, showed no significant effect. These conditions extracted 2.3 ± 0.2% of xylosaccharides (o.d.w.), representing 15.6 ± 0.3% of the xylosaccharides initially in the raw material.

Different precipitation conditions for xylosaccharides from alkaline extracts were studied using solvents. Ethanol was confirmed as an appropriate solvent for precipitating hemicelluloses from alkaline extraction. Lowering the pH before adding ethanol allows a fraction of lignin fragment separation, which would otherwise precipitate with ethanol addition. The highest precipitation yields and xylosaccharide proportions were achieved by adjusting the pH to 7 and working with one volume of ethanol per volume of liquor.

## Figures and Tables

**Table 1:** Chemical composition of *Eucalyptus* pinchips used as raw material.

Component	X ± 2σ ^1^ (%)
Glucan	41.9 ± 0.6
Xylan	14.5 ± 0.4
Arabinan	1.6 ± 0.3
Acid soluble lignin	2.8 ± 0.1
Acid insoluble lignin	21.9± 0.9
Acetyl groups	3.1 ± 0.1
Ethanol/water extractives	3.4 ± 0.2
Ash content	0.7 ± 0.1

^1^ Average percentage values of four replicates ± standard deviation, dry basis.

**Table 2:** Extraction yield (%), xylosaccharides content, and absorbance at 280 nm of the extraction liquors obtained for the 3^3^ experimental design.

Real Variables	Coded Variables	Extraction Yield (% o.d. Biomass)	Xylosaccharides Content (% o.d.Biomass)	Absorbance at 280 nm (Dilution 1/1500)
105 °C, 6.7 %NaOH, 45 min	−1, −1, −1	9.4 ± 0.5	0.7 ± 0.1	0.247 ± 0.012
105 °C, 6.7 %NaOH, 90 min	−1, −1, 0	12.8 ± 0.5	0.9 ± 0.1	0.446 ± 0.020
105 °C, 6.7 %NaOH, 135 min	−1, −1, +1	14.2 ± 0.6	1.2 ± 0.1	0.486 ± 0.019
105 °C, 11.7 %NaOH, 45 min	−1, 0, −1	16.3 ± 0.5	1.5 ± 0.1	0.284 ± 0.016
105 °C, 11.7 %NaOH, 90 min	−1, 0, 0	19.4 ± 0.7	1.7 ± 0.2	0.618 ± 0.010
105 °C, 11.7 %NaOH, 135 min	−1, 0, +1	21.0 ± 0.6	1.7 ± 0.1	0.707 ± 0.020
105 °C, 16.7 %NaOH, 45 min	−1, +1, −1	18.4 ± 0.5	2.3 ± 0.1	0.302 ± 0.014
105 °C, 16.7 %NaOH, 90 min	−1, +1, 0	21.7 ± 0.5	1.9 ± 0.1	0.729 ± 0.015
105 °C, 16.7 %NaOH, 135 min	−1, +1, +1	21.1 ± 0.7	1.9 ± 0.2	0.651 ± 0.012
135 °C, 6.7 %NaOH, 45 min	0, −1, −1	13.3 ± 0.5	1.3 ± 0.1	0.365 ± 0.016
135 °C, 6.7 %NaOH, 90 min	0, −1, 0	14.6 ± 0.4	1.2 ± 0.2	0.473 ± 0.020
135 °C, 6.7 %NaOH, 135 min	0, −1, +1	15.9 ± 0.6	1.1 ± 0.1	0.497 ± 0.023
135 °C, 11.7 %NaOH, 45 min	0, 0, −1	21.7 ± 0.7	1.5 ± 0.2	0.675 ± 0.016
135 °C, 11.7 %NaOH, 90 min	0, 0, 0	23.5 ± 0.6	1.6 ± 0.2	0.347 ± 0.014
135 °C, 11.7 %NaOH, 135 min	0, 0, +1	25.4 ± 0.7	1.8 ± 0.2	0.394 ± 0.020
135 °C, 16.7 %NaOH, 45 min	0, +1, −1	20.9 ± 0.4	2.2 ± 0.1	0.686 ± 0.016
135 °C, 16.7 %NaOH, 90 min	0, +1, 0	23.8 ± 0.4	2.2 ± 0.2	0.776 ± 0.018
135 °C, 16.7 %NaOH, 135 min	0, +1, +1	25.7 ± 0.6	2.2 ± 0.2	0.786 ± 0.021
155 °C, 6.7 %NaOH, 45 min	+1, −1, −1	17.6 ± 0.6	2.0 ± 0.2	0.486 ± 0.013
155 °C, 6.7 %NaOH, 90 min	+1, −1, 0	17.9 ± 0.4	2.1 ± 0.1	0.507 ± 0.013
155 °C, 6.7 %NaOH, 135 min	+1, −1, +1	19.4 ± 0.5	2.2 ± 0.2	0.498 ± 0.020
155 °C, 11.7 %NaOH, 45 min	+1, 0, −1	24.0 ± 0.6	2.0 ± 0.1	0.810 ± 0.021
155 °C, 11.7 %NaOH, 90 min	+1, 0, 0	25.6 ± 0.6	2.1 ± 0.2	0.803 ± 0.018
155 °C, 11.7 %NaOH, 135 min	+1, 0, +1	27.6 ± 0.7	2.2 ± 0.2	0.887 ± 0.015
155 °C, 16.7 %NaOH, 45 min	+1, +1, −1	35.1 ± 0.6	2.1 ± 0.1	1.085 ± 0.018
155 °C, 16.7 %NaOH, 90 min	+1, +1, 0	34.9 ± 0.5	2.3 ± 0.1	0.995 ± 0.020
155 °C, 16.7 %NaOH, 135 min	+1, +1, +1	38.8 ± 0.6	2.5 ± 0.2	1.208 ± 0.020

**Table 3:** Molecular weight distribution of the precipitates obtained after 1 volume of ethanol addition at different liquor pH. Total indicates the mean molecular weight obtained considering the contribution of the peaks observed in the sample.

pH	Molecular Weight Distribution				
	#	Mn (Da)	Mw (Da)	Mw/Mn	Relative Area (%)
12.4	Total	4080	33,243	3.6	
	1	143,868	154,141		14.8
	2	29,864	30,428		16.0
	3	11,407	11,982		42.5
	4	1325	1615		1.2
9.1	Total	8011	16,873	1.2	
	1	16,062	18,433		90.7
	2	1358	1633		9.3
7.2	Total	5255	16,147	3.1	
	1	42,937	43,899		10.0
	2	22,815	24,378		47.8
	3	1420	1930		42.2
5.1	Total	8310	18,658	2.2	
	1	27,146	28,031		17.0
	2	11,569	12,574		56.5
	3	1309	1699		26.5
1.9	Total	21,038	26,569	1.3	
	1	44,481	46,150		17.2
	2	18,960	22,497		82.8

#: Peak number, Mn: Number average molecular weight; Mw: Weight average molecular weight.

**Table 4:** Molecular weight distribution of the obtained precipitates using different ethanol-to-liquor volume ratios at pH 7.

Ethanol-to-Liquor Volume Ratio	Molecular Weight Distribution				
	#	Mn (Da)	Mw (Da)	Mw/Mn	%
1:1	Total	4908	19,340	3.9	
	1	21,401	24,798		76.3
	2	1407	1718		26.7
2:1	Total	2885	14,838	5.1	
	1	21,552	24,436		58.0
	2	1315	1600		42.0
3:1	Total	2322	10,894	4.7	
	1	26,575	27,582		34.1
	2	1577	2262		65.9
4:1	Total	2171	11,167	5.1	
	1	23,606	25,559		39.6
	2	1361	1740		60.4

#: Peak number, Mn: Number average molecular weight; Mw: Weight average molecular weight.

## Data Availability

Data are contained within the article.
